# Comparative results of percutaneous and open surgery for trigger fingers: a propensity score analysis

**DOI:** 10.3389/fsurg.2025.1509292

**Published:** 2025-03-06

**Authors:** Praphan Chanthanapodi, Sasithorn Aodsup

**Affiliations:** Orthopaedic Department, Somdejphrajaotaksin Maharaj Hospital, Tak, Thailand

**Keywords:** trigger finger, percutaneous release, open release, nerve injury, recurrence, propensity score

## Abstract

**Background and objectives:**

Trigger finger is a common hand condition characterized by the locking of a digit, often requiring surgical intervention when conservative treatments fail. This study aimed to compare the outcomes of a modified percutaneous release technique with those of traditional open release surgery.

**Materials and methods:**

A retrospective cohort study was conducted on 245 patients (287 digits), of which 161 digits underwent open release and 126 underwent percutaneous release. A modified technique for percutaneous release was described. Propensity score matching was used to balance the data. Cox regression and Laplace regression were applied to analyze the hazard ratio and median survival time for pain relief and time to return to work. Adverse events were also reported.

**Results:**

The duration of pain relief in the percutaneous release group was shorter than that in the open release group (hazard ratio = 1.73, 95% CI: 0.98–3.06; *p* = 0.057). Fifty percent of patients in the percutaneous release group experienced pain relief within two days, compared to seven days in the open release group (*p* = 0.003). Time to return to work was significantly shorter in the percutaneous release group than in the open release group (hazard ratio = 2.93, 95% CI: 2.08–4.13; *p* < 0.001). Fifty percent of patients in the percutaneous release group returned to work within three days, compared to 15 days in the open release group (*p* < 0.001). Three digits (2.4%) required conversion to open release due to the failure of percutaneous release. No nerve injuries or recurrences were observed at a follow-up of 42.2 ± 2.2 months.

**Conclusions:**

Percutaneous release resulted in an earlier return to work and a high success rate (97.6%) with no nerve injuries or recurrences over 42 months. Despite a 2.4% failure rate, careful technique minimized complications. Further randomized trials are needed to confirm these findings and optimize patient selection.

## 1 Introduction

Trigger finger, or stenosing tenosynovitis, was first described by Alphonse Nota in 1850 (1). It is a common hand condition characterized by “triggering” or locking of the affected digit. This occurs when inflammation of the flexor tendon sheath leads to fibrocartilage metaplasia, most commonly at the A1 pulley, restricting tendon mobility and impairing hand function. Several factors, including synovial proliferation and flexor sheath fibrosis, have been linked to triggering; however, no consensus exists regarding its exact etiology (2).

For patients unresponsive to conservative treatments, surgical intervention may be necessary. Although open release remains the traditional method, percutaneous release, first introduced by Eastwood et al., has gained popularity due to its minimally invasive nature (3). This approach has been shown to reduce pain duration and expedite return to work (4). Various devices and techniques have been developed to improve treatment outcomes (5–7), but they may increase costs and limit availability. Consequently, needles of various sizes (3, 8–10) remain widely preferred for their convenience.

Since percutaneous release is a blind procedure, there is a risk of adverse events, including procedural failure, nerve injury, and recurrence, which may occur even years after surgery. A meta-analysis by Fiorini et al. (11) found that percutaneous and open surgeries have comparable symptom resolution rates; however, there is insufficient evidence to determine the superiority of one technique over the other in terms of pain reduction, recurrence, adverse events, or neurovascular injury. To address these limitations and improve outcomes, a modified technique has been developed to simplify the procedure and enhance safety. This study aims to compare the short- and long-term outcomes of a modified percutaneous release technique with open-release surgery, specifically analyzing differences in time to pain relief, time to return to work, and associated adverse events.

## 2 Materials and methods

This study was a retrospective observational cohort study. The protocol was approved by the institutional review board (IRB), approval number COA 1/2023. The study included consecutive patients (aged 18 years and older) who underwent surgical release for trigger finger at Somdejphrajaotaksin Maharaj Hospital, Thailand, between January 2018 and December 2023, following the failure of conservative treatment.

Before surgery, all participants received comprehensive information about the planned surgical procedures (open or percutaneous release) from their attending physicians and provided written informed consent. Both types of surgery were performed by board-certified orthopedic surgeons. The inclusion criteria for surgery required either a history of unsuccessful conservative treatment or the patient's preference for surgical intervention. Patients were excluded from percutaneous release if they had a history of prior surgery, Dupuytren's contracture, scleroderma, scar contracture, or any other condition significantly impairing skin flexibility.

### 2.1 Percutaneous surgical technique

**Figure 1:**
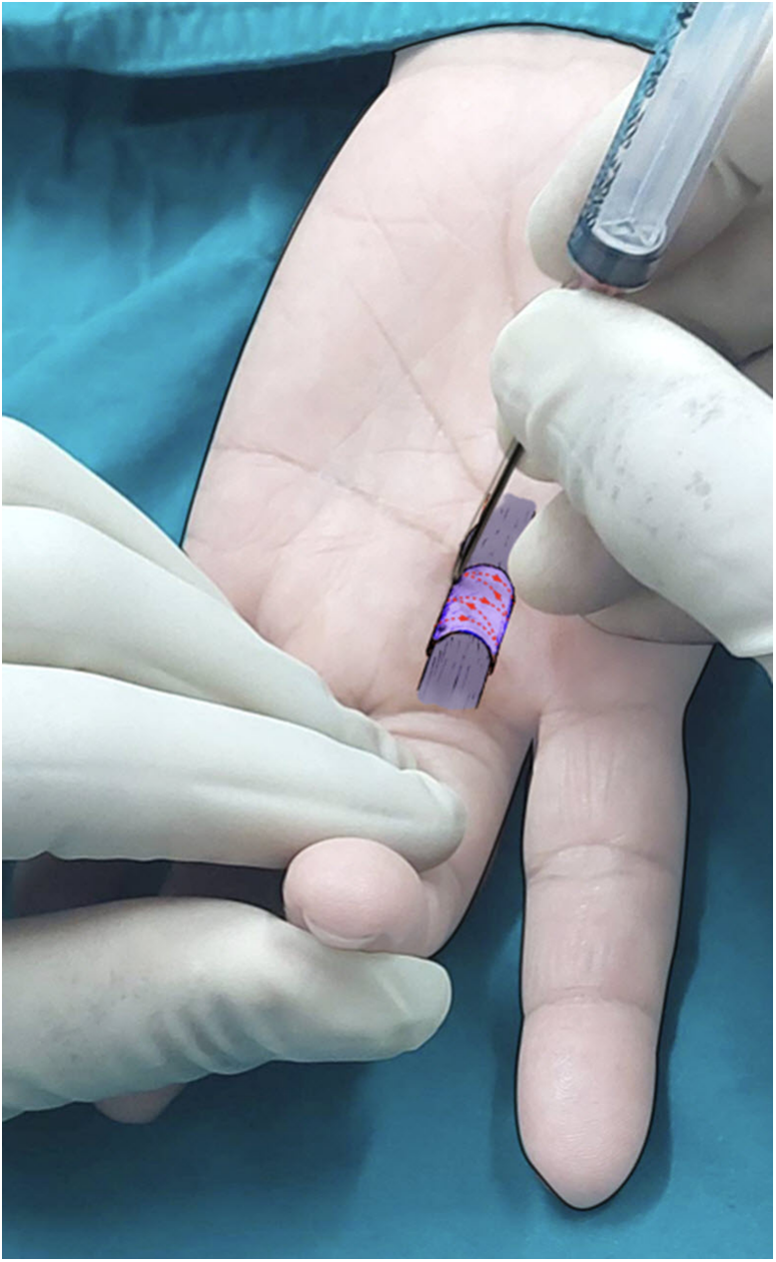
Confirmation of the outline and midline of the A1 pulley in the flexed position by palpating with the needle tip in a zigzag pattern.

1. Patient Positioning: The patient was positioned opposite the surgeon with the affected hand supinated and parallel to the floor. For thumb procedures, the patient sat to the right of the surgeon with the hand resting on the table edge in supination. The surgical site was prepared with chlorhexidine-alcohol.
2. Identification of the midline of the A1 Pulley: Determined the midline of the A1 pulley on the skin surface.
3. Anesthesia: Local anesthesia was injected at the center of the midline or slightly proximal to it.
4. Needle Insertion: Inserted an 18-gauge needle at the designated injection site. Ensured the needle bevel was aligned with the syringe flange to visualize its orientation beneath the skin. Using the dominant hand (right hand in right-handed individuals), grasped the needle and syringe, aligning the bevel with the long axis of the A1 pulley. The non-dominant hand held the digit in flexion to relax the skin. Maintained the needle bevel parallel to the longitudinal axis of the digit throughout the procedure.
5. Confirmation of the outline and the midline of the A1 pulley: Palpated the A1 pulley with the needle tip, moving it in a zigzag pattern from proximal to distal across the midline. The midline on the A1 pulley should have been aligned with the skin-marked midline and repeated this process until the midline is confidently identified (Figure 1).

Steps 4 and 5 were modified from a previously published technique (3).

**Figure 2:**
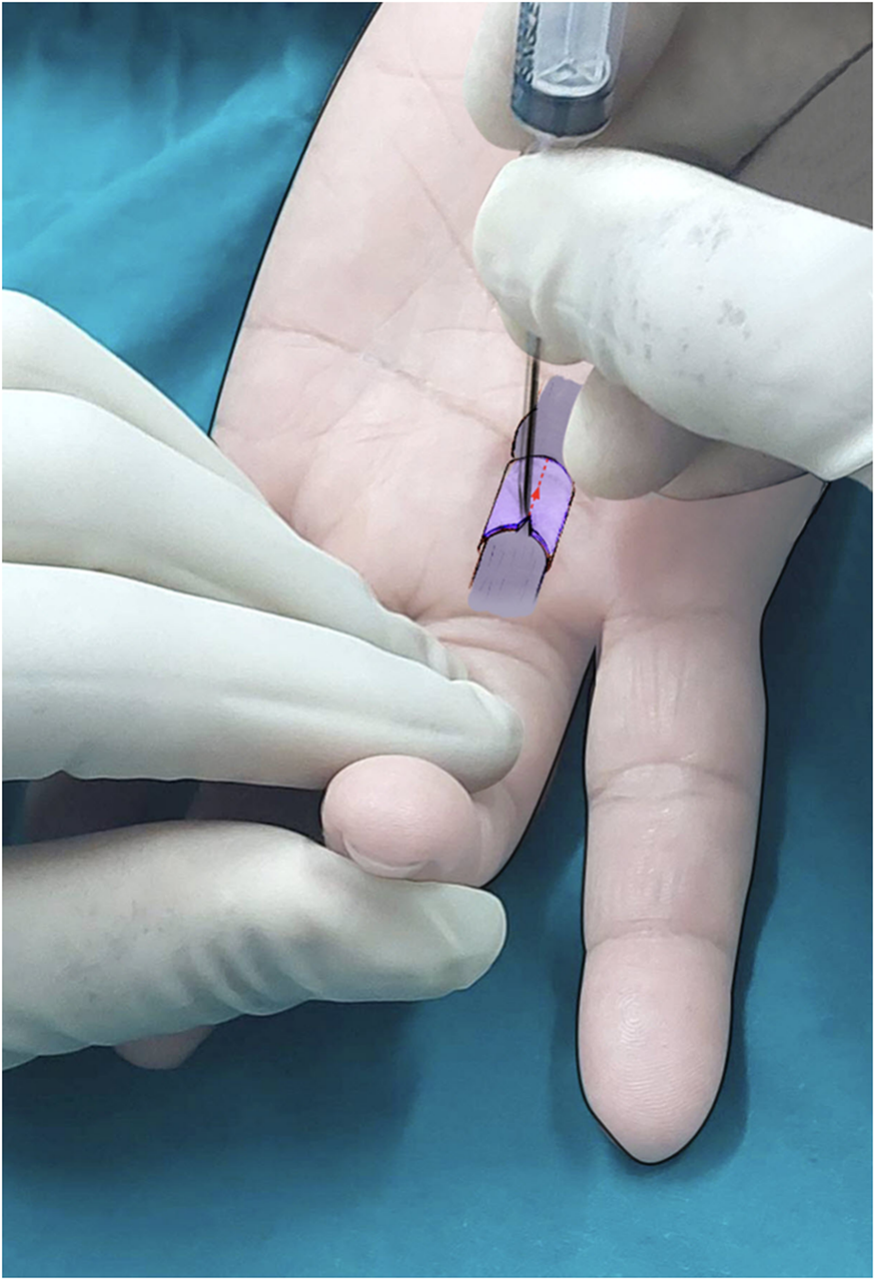
A complete release of the A1 pulley along the central longitudinal axis using the needle bevel, starting at the distal end and progressing proximally until the grating sensation ceases. The patient's finger is kept in the flexed position throughout the release procedure. *The hand in both pictures is mine, so I did not request consent*.

6. Incision of the A1 Pulley: Incised the A1 pulley longitudinally, beginning at the distal end and progressing proximally until the grating sensation ceased (Figure 2). To prevent lateral deviation from the midline, maintain the needle perpendicular to the palmar plane throughout the procedure. For thumb procedures, exercise caution to avoid inadvertently sectioning the proximal border (as indicated by a grating sound), which might risk injury to the radial digital nerve.
7. Needle Removal and Function Testing: Removed the needle and had the patient perform flexion and extension movements several times during wound compression for approximately 10 min
8. Revision of Incision: If trigger finger symptoms persist, repeat the release procedure, using the previously marked skin as a reference. Incomplete incisions frequently occur at the proximal end of the A1 pulley. For patients with a thick A1 pulley, such as in the middle finger, or limited skin elasticity, consider adjusting the second needle insertion point slightly distal or proximal to the first insertion point. If necessary, this step can be converted to an open release.
9. Post-operative Dressing: Applied a plaster strip for 6 h.
10. Post-operative Activity: Daily living activities were initiated on the same day, and patients could gradually resume them as their pain diminished

### 2.2 Outcome measurement and definitions

The primary outcomes assessed in this study were the time to pain relief and the time to return to work. The follow-up period was restricted to 30 days, and patients who had not achieved pain relief or resumed work within this timeframe were censored. Time to pain relief was defined as the duration from surgery to the patient's self-reported onset of significant pain reduction (12). Similarly, time to return to work was defined as the period from surgery until the individual resumed occupational duties.

For long-term outcomes, including nerve injury and recurrence (key concerns associated with percutaneous release), telephone follow-ups were conducted in June and July 2024. During this period, patients were contacted via telephone and asked about both primary and long-term outcomes, including the following questions:

• The number of days post-surgery until they consistently experienced a significant reduction in pain—both at rest and during activity—for at least 24 consecutive hours without requiring analgesics.
• The recovery period (in days) needed to resume normal daily activities or occupational duties without experiencing pain or movement restrictions.
• The presence of numbness in the finger after undergoing trigger finger release surgery.
• The reappearance of triggering symptoms after initial resolution following surgery.

The interview process was validated through pre-testing and interviewer training. Additionally, interviewers were blinded to the treatment received by the patients and underwent training to standardize their interviewing techniques.

Information collected from electronic medical records included patient demographics (such as gender and age), digit type, symptom duration, comorbidities (e.g., diabetes mellitus and carpal tunnel syndrome), history of steroid injections, Quinnell grading system (13), and failure of percutaneous release. Percutaneous release failure was identified when the procedure required conversion to an open release due to persistent finger-catching or triggering symptoms, despite the complete resolution of the grating sensation during the percutaneous release.

### 2.3 Statistical analysis

Continuous data were analyzed using Student's *t*-test, while ordinal and binary data were evaluated using the exact probability test. Standardized differences (STD) were computed, with a threshold of >0.10 indicating a significant difference between groups. Missing data were addressed through mode imputation.

Since this study employed an observational design, the two intervention methods were not randomly assigned, leading to potential confounding by indication and contraindication, which could have distorted the true associations between clinical endpoints. To address this, propensity score matching was applied—a standard tool for non-randomized or observational studies (14)—to enhance comparability between groups. A multivariable logistic regression model was used to derive propensity scores from pretreatment characteristics that influenced treatment selection. STD values were computed, with a threshold of >0.10 indicating a significant difference between groups (15). Propensity score matching was then performed to balance these variables across treatment groups before the final analysis. For the final analysis, hazard ratios for time to pain relief and time to return to work were calculated using Cox proportional hazards regression. Median survival time was analyzed using Laplace regression.

The sample size was retrospectively calculated based on the hazard ratio, with a desired power of 0.80 and a two-sided alpha level of 0.05. Statistical analyses were performed using Stata/MP 16.1 (Stata 12.1 for sample size calculation) software (StataCorp LP, College Station, TX, USA), and statistical significance was set at *p* < 0.05.

## 3 Results

**Figure 3:**
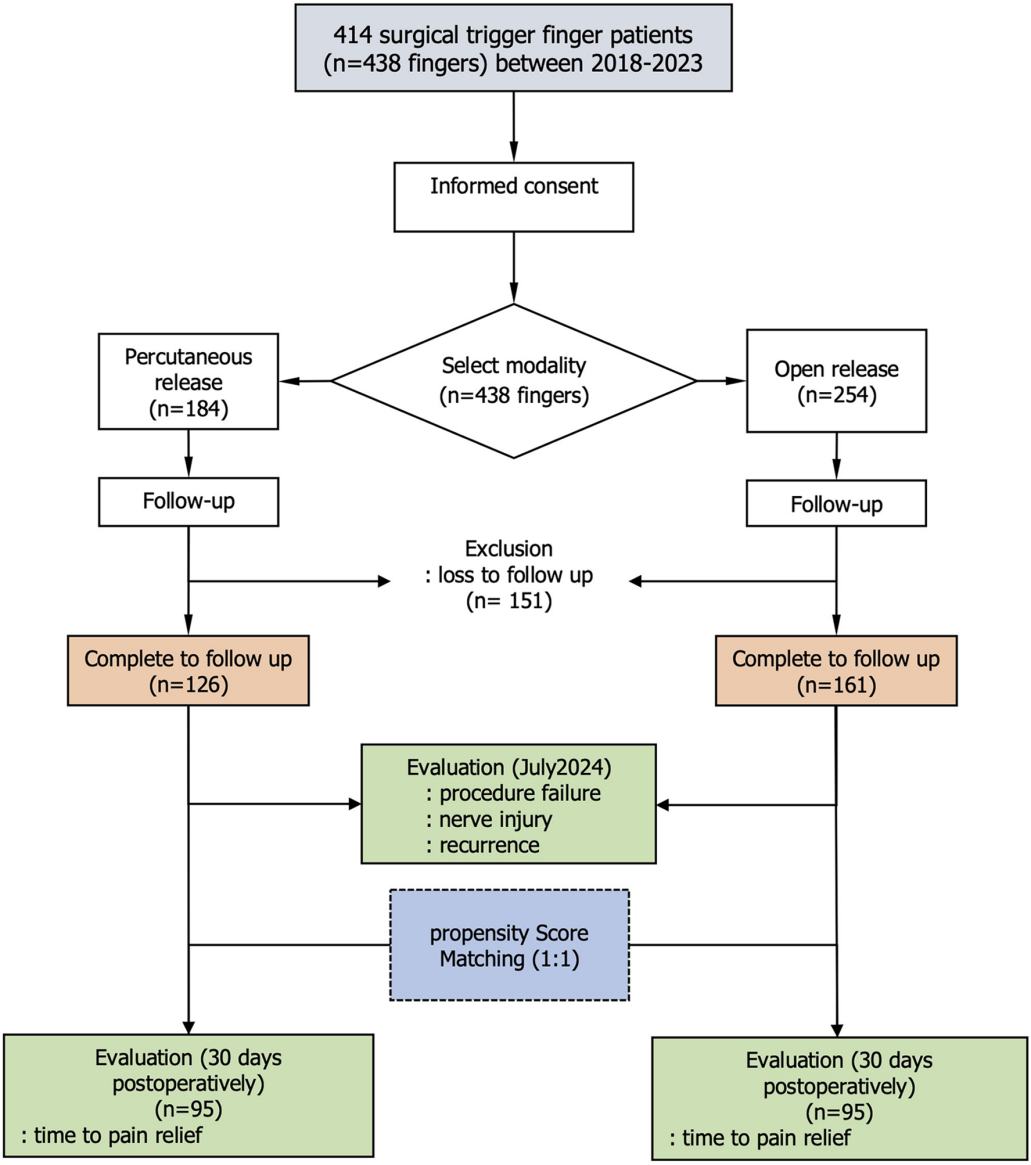
Study flow diagram.

This study included 414 patients (438 digits) who underwent either open or percutaneous release surgery between 2018 and 2023. Of these, 245 patients (287 digits) completed the study, with 161 patients undergoing open release and 126 patients undergoing percutaneous release (Figure 3). A total of 169 out of 414 patients (40.8%) were excluded from the study because they could not be contacted by phone for an interview. Data were available for both 30-day postoperative and long-term follow-ups. The mean long-term follow-up duration was 43.8 ± 2.9 months for the open-release group and 42.2 ± 2.2 months for the percutaneous-release group (*p* = 0.531). Missing data were observed for the following variables: diabetes mellitus (8, 2.8%), carpal tunnel syndrome (22, 7.7%), Quinnell grading (149, 51.9%), and number of steroid injections (33, 11.5%). All missing values were addressed using mode imputation. Gender, digit type, Quinnell grade, symptom duration, and number of steroid injections exhibited standardized differences (STDs) greater than 10%. Furthermore, three of the eight variables—gender, Quinnell grade, and number of steroid injections—showed statistically significant differences (*p* < 0.05) between the groups (Table 1).

**Table 1:** Patient characteristics.

Characteristics	Percutaneous	Open release	^†^STD	*p*-value
	*N* = 126	*N* = 161		
	(*n* %)	(*n* %)		
Gender				
Female	92 (73.0)	141 (87.6)	0.37	0.002
Male	34 (27.0)	20 (12.4)		
Age (mean ± SD)	54.7 ± 8.3	55.0 ± 9.2	0.03	0.793
Diabetes mellitus	32 (25.4)	36 (22.4)	0.07	0.578
Carpal tunnel syndrome	17 (13.5)	23 (14.3)	0.02	0.866
Digit type				
Thumb	53 (42.1)	58 (36.0)	0.37	0.059
Index	15 (11.9)	17 (10.6)		
Middle	33 (26.2)	63 (39.1)		
Ring	24 (19.0)	18 (11.2)		
Little	1 (0.8)	5 (3.1)		
Quinnell grade				
1	5 (4.0)	4 (2.5)	0.40	0.008
2	92 (73.0)	142 (88.2)		
3	11 (8.7)	7 (4.3)		
4	18 (14.3)	8 (5.0)		
Duration (month), median [IQR]	3.0[1, 3.4]	3.4[1, 5.0]	0.15	0.217
Number of steroid injections				
0	104 (82.5)	116 (72.0)	0.38	0.015
1	12 (9.5)	37 (23.0)		
2	7 (5.6)	5(3.1)		
3	3(2.4)	3(1.9)		

^†^STD, standardized difference.

**Figure 4:**
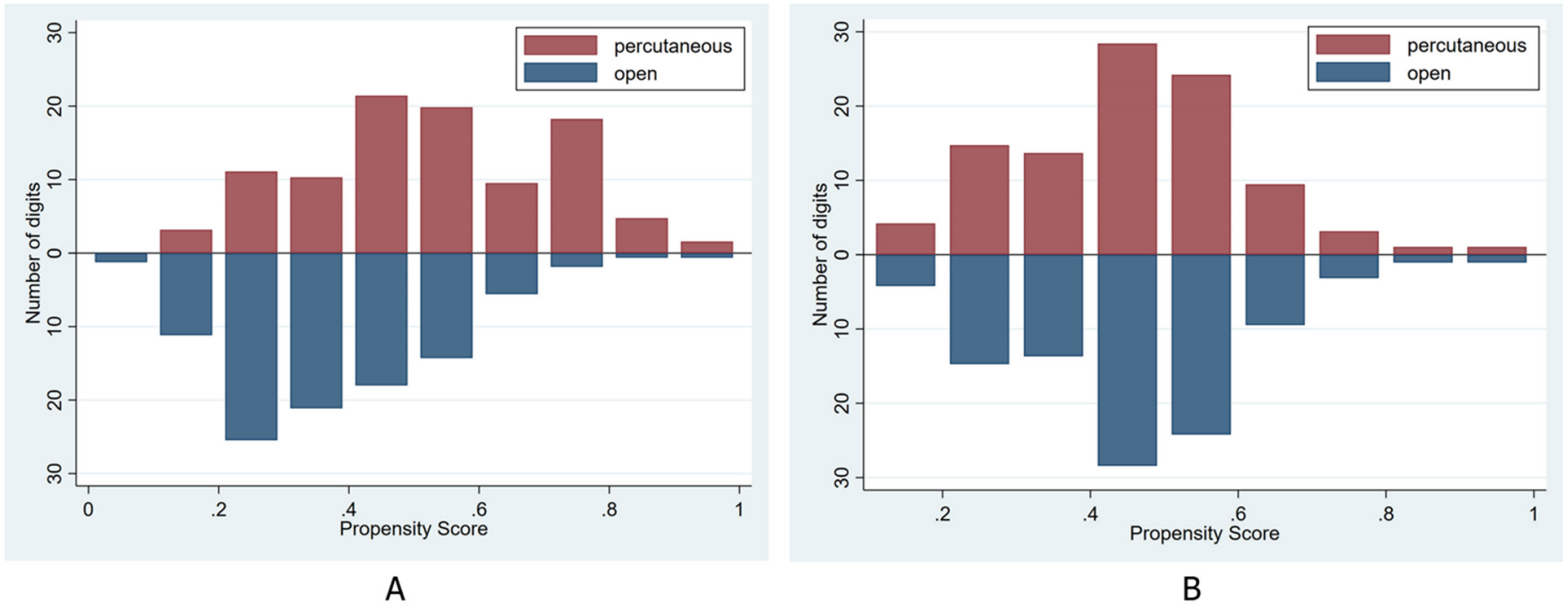
Histograms illustrating the distribution of propensity scores in both groups—before propensity score matching **(A)** and after propensity score matching **(B)**.

The propensity scores were calculated using a multivariable logistic model that included gender, age, comorbidities (diabetes mellitus and carpal tunnel syndrome), digit type, Quinnell grade, symptom duration, and the number of steroid injections (Table 2). The mean propensity score was significantly different before matching (0.37 ± 0.16 vs. 0.52 ± 0.19, *p* < 0.001). The graph illustrates an imbalance in patient characteristics between the percutaneous and open groups (Figure 4A). After propensity score matching, the standardized difference (STD) for all eight variables—gender, age, diabetes mellitus, carpal tunnel syndrome, digit type, Quinnell grade, symptom duration, and number of steroid injections—was reduced to less than 10%. This corresponded to non-significant *p*-values (*p* ≥ 0.05) for each variable. The sample size was adjusted to 95 digits per group (Table 3). The mean propensity scores in each group became nearly equal (0.49 ± 0.04 vs. 0.50 ± 0.04, *p* = 0.305). The graph illustrates a balance in patient characteristics between the percutaneous and open groups (Figure 4B).

**Table 2:** Derivation of propensity score via multivariable logistic regression model.

Pre-treatment covariates	Coefficient	95% confidence interval	*p*-value
Gender	1.1081	0.4685, 1.7477	0.001
Age	−0.0055	−0.0335, 0.0224	0.698
Diabetes mellitus	0.1720	−0.4081, 0.7521	0.561
Carpal tunnel syndrome	0.2655	−0.4545, 0.9856	0.470
Digit type	−0.0886	−0.2988, 0.1216	0.409
Quinnell grade	0.5839	0.1940, 0.9739	0.003
Duration of symptom	−0.0435	−0.1116, 0.0245	0.210
Number of steroid injections	−0.1448	−0.5287, 0.2390	0.460
Constant(intercept)	−1.1332	−2.9521, 0.6855	0.222

**Table 3:** Patient characteristics (propensity score matched data).

Characteristics	Percutaneous	Open release	^†^STD	*p*-value
	*N* = 95	*N* = 95		
	(*n* %)	(*n* %)		
Gender				
Female	77 (81.1)	77 (81.1)	0.00	1.000
Male	18 (18.9)	18 (18.9)		
Age (mean ± SD)	54.6 ± 8.3	54.7 ± 9.8	0.01	0.930
Diabetes mellitus	22 (23.2)	18 (18.9)	0.10	0.594
Carpal tunnel syndrome	13 (13.7)	13 (13.7)	0.00	1.000
Digit type				
Thumb	38 (40.0)	40 (42.1)	0.07	0.989
Index	10 (10.5)	11 (11.6)		
Middle	30 (31.6)	29 (30.5)		
Ring	16 (16.8)	14 (14.7)		
Little	1 (1.1)	1 (1.1)		
Quinnell grade				
1	4 (4.2)	3 (3.2)	0.07	1.000
2	79 (83.2)	79 (83.2)		
3	5 (5.3)	5 (5.3)		
4	7 (7.4)	8 (8.4)		
Duration (month), median [IQR]	2.0[1, 5]	2.0[1, 4]	0.02	0.915
Number of steroid injections				
0	77 (81.1)	76 (80.0)	0.03	1.000
1	11 (11.6)	12 (12.6)		
2	5 (5.3)	5(5.3)		
3	2(2.1)	2(2.1)		

^†^STD, standardized difference.

A retrospective sample size calculation was performed based on a hazard ratio of 1.73 for pain relief, a pain relief prevalence of 0.97, a withdrawal rate of 34.4% (151 out of 438 digits), a power of 80%, a significance level of 0.05, and a two-sided test. The minimum required sample size was 164 digits (82 cases and 82 controls).

The hazard ratio for time to pain relief between the percutaneous and open groups was 1.73 (95% CI: 0.98–3.06; *p* = 0.057). The median time to pain relief was 2 days for the percutaneous group and 7 days for the open group (*p* = 0.003). The hazard ratio for time to return to work between the groups was 2.93 (95% CI: 2.08–4.13; *p* < 0.001). The median time to return to work was 3 days for the percutaneous group and 15 days for the open group (*p* = 0.003) (Table 4).

**Table 4:** Final results after propensity score matching.

Outcomes	Hazard ratio	Median survival time (day)		95% CI	*p*-value
		PCR^†^	Open		
The time to pain relief	1.73			0.98–3.06	0.057
		2	7		0.003
The time to return to work	2.93			2.08–4.13	<0.001
		3	15		<0.001

^†^PCR, percutaneous release.

Among the percutaneous procedures, failure of percutaneous release was observed in three digits (2.4%). No nerve injuries were identified. No recurrence was noted during long-term follow-up, with a follow-up time of 42.2 ± 2.2 months (Table 5).

**Table 5:** Postoperative adverse events (Pre-propensity score matching).

Adverse events	Percutaneous	Open release
	*N* = 126	*N* = 161
	*n* (%)	*n* (%)
Procedure failure	3 (2.4)	0 (0)
Nerve injury	0 (0)	–
Recurrence	0 (0)	0 (0)

## 4 Discussion

**Figure 5:**
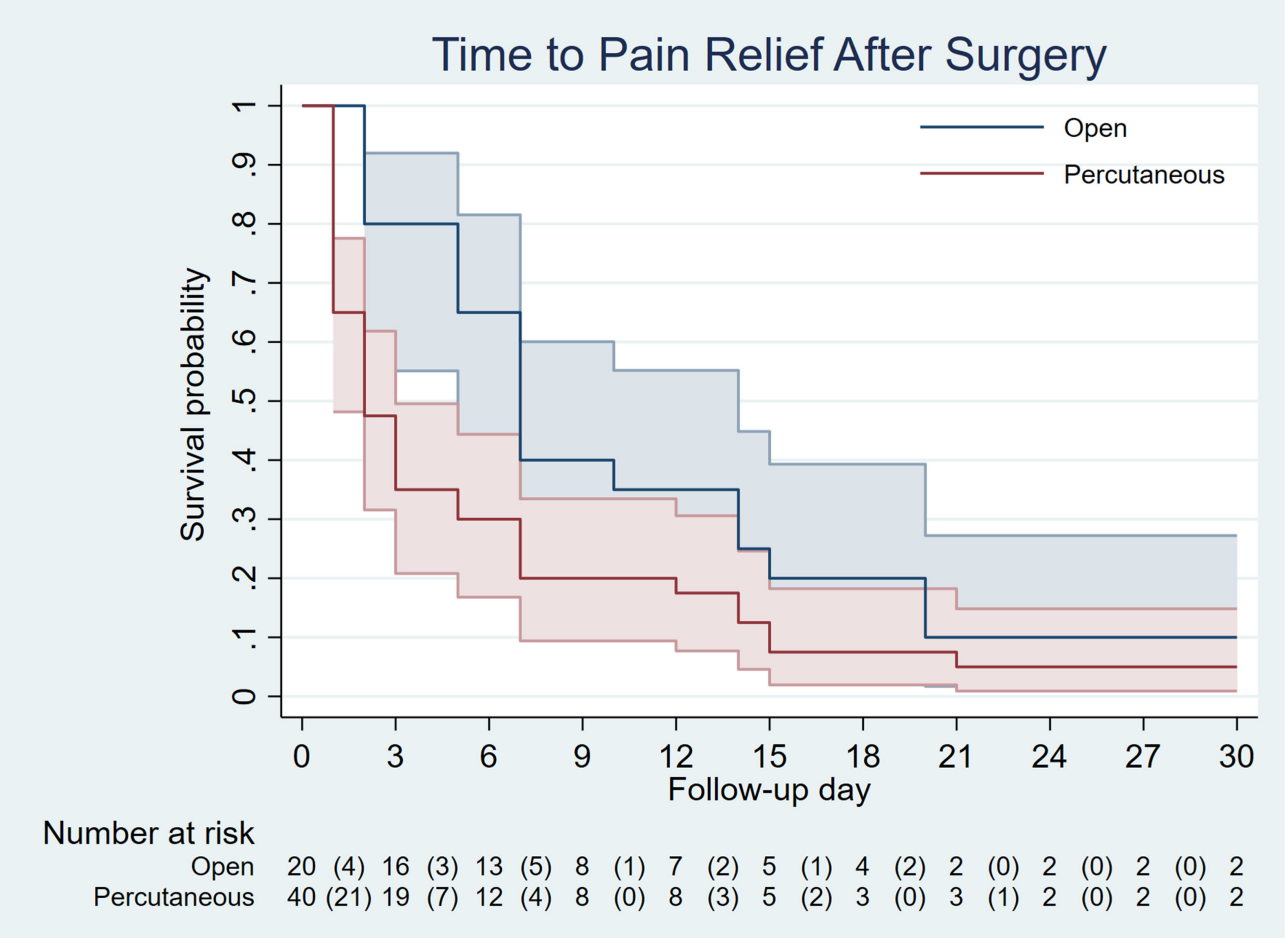
Time to pain relief after surgery.

**Figure 6:**
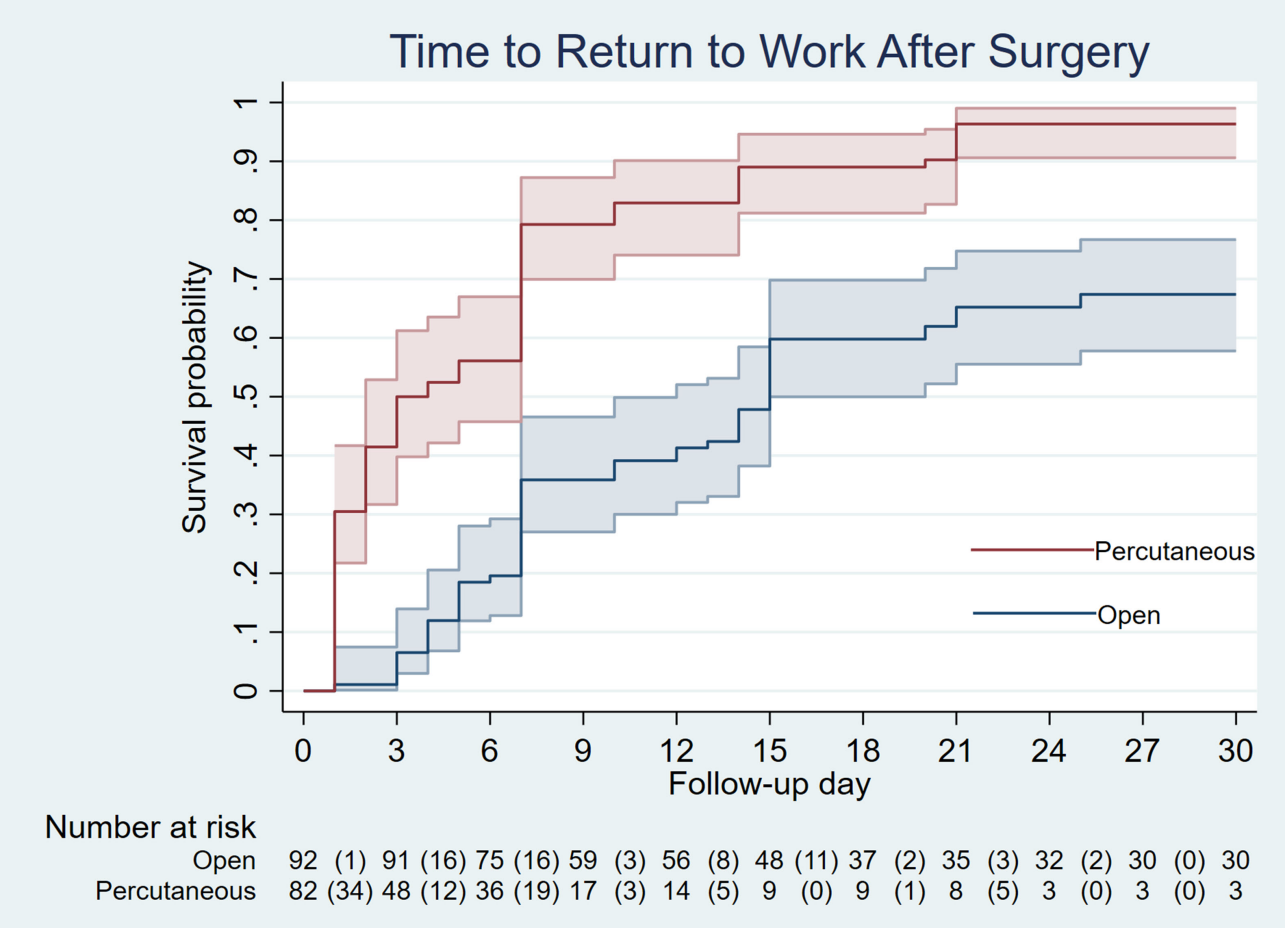
Time to return to work after surgery.

Following propensity score matching, the standardized difference for all covariates was less than 10%, and all *p*-values were ≥ 0.05 (Table 3), indicating that the distribution of covariates was well-balanced between the two groups, similar to what would be expected in a randomized controlled trial. After data balancing, the time to pain relief and return to work were analyzed. The results indicated that 50% of patients experienced pain relief earlier in the percutaneous release group (2 days) compared to the open release group (7 days), with nearly all patients in both groups achieving pain relief within three weeks (Figure 5). However, the hazard ratio between the groups did not show a significant difference. Therefore, the advantage of percutaneous release in reducing the time to pain relief remains inconclusive. Moreover, percutaneous release led to a significantly faster return to work, with 50% of patients resuming work within 3 days, compared to 15 days in the open release group. The hazard ratio indicated that patients in the percutaneous group returned to work 2.9 times faster than those in the open group, with statistical significance. Additionally, while nearly all patients in the percutaneous group had resumed work by the third postoperative week, a substantial proportion of patients in the open group remained unable to do so (Figure 6). These findings suggest that the minimally invasive nature of percutaneous release, which reduces tissue trauma and accelerates wound healing, contributes to an earlier return to work.

Nerve injury, particularly in the thumb, is a potential complication of percutaneous release. Although F. Guler et al. (16) reported incidence rates as high as 5.7%, most studies have shown lower rates or no cases (17–19). In this study of 126 digits, no nerve injuries were observed. This may be attributed to the modified surgical technique, which involved placing the thumb in a flexed position to reduce tension on the digital nerve. Careful distal-to-proximal release, stopping immediately upon complete release, as indicated by the absence of grating, helped avoid inadvertent injury to the radial branch of the digital nerve. For other digits, identifying the longitudinal midline of the pulley and performing the release in a flexed position further reduced the risk of nerve injury. These findings suggest that meticulous surgical technique, including proper finger positioning and careful dissection, significantly minimizes the risk of nerve injury during percutaneous release. Furthermore, placing the digit in flexion resulted in skin laxity, facilitating smooth needle movement and allowing for easier A1 pulley release. This technique minimizes the risk of injuring surrounding tissues and may also reduce the incidence of chronic pain. However, most reports advocate for a hyperextension position, as described by Eastwood et al. (3), with the expectation of maximizing the distance between the needle and the neurovascular bundle.

Recurrence is a significant complication associated with percutaneous release, often linked to incomplete initial release and subsequent fibrous tissue formation. Huang H-K et al. (20) reported a recurrence rate of 8.8% with percutaneous release, compared to 3.4% with open release. Aksoy A et al. (21) found that recurrence can occur up to three years postoperatively, particularly in the ring and middle fingers, highlighting the importance of long-term follow-up. In our study, with a mean follow-up of 42 months (range: 7–77 months), no recurrence was observed in the percutaneous group. This may be attributed to factors such as complete release, indicated by smooth needle tip sensation (absence of grating sensation) and the resolution of triggering symptoms during active motion testing for at least 10 min post-release. Therefore, selecting patients with triggering symptoms (Quinnell grades 2–4) as a criterion for postoperative assessment is essential. However, five patients in this study with initial Quinnell grade 1 symptoms, followed for 64.2–73.9 months, also showed no recurrence. Although no studies have definitively confirmed the absence of recurrence in Quinnell grade 1 patients after percutaneous release, further research is needed.

The most common complication associated with percutaneous release is the need for conversion to open release due to a failed initial release**.** As reported in a case series by Qureshi et al. (22), three digits (9.3%)**,** Werthel JD et al. (22)**,** eight digits (6%)**,** and Tawfik et al. (23), three digits (15%) required conversion, emphasizing the importance of the surgeon's clinical judgment in determining when to switch to an open approach, particularly if persistent triggering occurs despite the absence of a grating sensation. This decision aims to prevent potential trauma to surrounding structures and mitigate the risk of further adverse events. In this study, failure occurred in three digits (2.4%), all of which were the middle or ring finger. Intraoperative findings revealed incomplete release of the proximal portion of the A1 pulley in all three cases. After confirming complete release, triggering symptoms resolved in all affected digits.

The overall success rates for percutaneous release varied widely, ranging from 82.6% (24) to 100% (25, 26). In this study, a success rate of 97.6% (123/126 digits) was achieved. We attributed this high success rate and the overall safety of the procedure to several key factors:

• Precise identification of the A1 pulley by palpating with the needle tip before incision,
• The degree of skin laxity, which influences the ease of dissection,
• The ability to accurately assess the grating sensation.

Consequently, a learning curve is likely to play a significant role in improving the success rate of percutaneous release (27).

This study has several limitations. First, as this research is a retrospective observational study, confounders due to indications and contraindications are inevitable. Patients who underwent open release may have had different baseline characteristics than those who underwent percutaneous release due to factors such as physician preference, patient comorbidities, or the severity of their condition. These imbalances, as demonstrated in Table 1, could potentially confound the observed outcomes and make it difficult to draw causal inferences. However, we used propensity score matching to address this limitation by reducing confounding between the control and study groups. Second, approximately 40.8% of patients were excluded from the study due to loss to follow-up by phone. However, after matching, the remaining participants still had more than 80% power to differentiate the primary outcome. Third, data on time to pain relief and return to work were primarily collected through telephone interviews, which may be subject to recall bias. Fourth, propensity score matching can lead to a reduction in sample size due to the exclusion of certain patients during the matching process. The selection of variables for matching was based on availability and clinical relevance. However, the exclusion of crucial variables, such as surgeon expertise, from the propensity score model may have limited the effectiveness of the matching process and potentially affected the robustness of the findings. Due to patient preference in choosing either open or percutaneous release, selection bias may have occurred. Further research using randomized controlled trials (RCTs) with a larger and more diverse population is warranted to confirm these findings, validate their generalizability, and address the limitations of this study. These efforts will contribute to improved clinical decision-making and the development of optimized treatment strategies for better patient care.

## 5 Conclusions

Percutaneous release demonstrated an earlier return to work compared to open release, with a high success rate (97.6%) and no observed nerve injuries or recurrence over a 42-month follow-up. Despite a 2.4% failure rate requiring conversion, meticulous surgical technique minimized complications. These findings support percutaneous release as an effective and minimally invasive alternative. Future randomized controlled trials are needed to validate these results and optimize patient selection to ensure improved outcomes in trigger finger treatment.

## Data Availability

The original contributions presented in the study are included in the article/Supplementary Material, further inquiries can be directed to the corresponding author.
